# Conditioned medium derived from rat amniotic epithelial cells confers protection against inflammation, cancer, and senescence

**DOI:** 10.18632/oncotarget.9694

**Published:** 2016-05-29

**Authors:** Clara Di Germanio, Michel Bernier, Michael Petr, Mauro Mattioli, Barbara Barboni, Rafael de Cabo

**Affiliations:** ^1^ Faculty of Veterinary Medicine, University of Teramo, Teramo, Italy; ^2^ Translational Gerontology Branch, National Institute on Aging, NIH, Baltimore, MD, USA; ^3^ Istituto Zooprofilattico Sperimentale dell'Abruzzo e del Molise “G. Caporale”, Teramo, Italy

**Keywords:** amniotic epithelial cells, inflammation, tumorigenesis, senescence, SASP, Gerotarget

## Abstract

Amniotic epithelial cells (AECs) are a class of fetal stem cells that derives from the epiblast and resides in the amnion until birth. AECs are suitable candidates for regenerative medicine because of the ease of collection, their low immunogenicity and inability to form tumors after transplantation. Even though human AECs have been widely investigated, the fact remains that very little is known about AECs isolated from rat, one of the most common animal models in medical testing. In this study, we showed that rat AECs retained stemness properties and plasticity, expressed the pluripotency markers Sox2, Nanog, and Oct4 and were able to differentiate toward the osteogenic lineage. The addition of conditioned medium collected from rat AECs to lipopolysaccharide-activated macrophages elicited anti-inflammatory properties through a decrease of *Tnfa* expression and slowed tumor cell proliferation *in vitro* and *in vivo*. The senescence-associated secretory phenotype was also significantly lower upon incubation of senescent human IMR-90 fibroblast cells with conditioned medium from rat AECs. These results confirm the potential of AECs in the modulation of inflammatory mechanisms and open new therapeutic possibilities for regenerative medicine and anti-aging therapies as well.

## INTRODUCTION

With the increase of the aging population and incidence of age-related diseases, regenerative medicine is becoming a promising strategy for healthspan and lifespan extension. Stem cell-based therapy is gaining greater interest in the field of regenerative medicine, as it allows the development of new clinical applications aimed at restoring tissue and organ function [1, 2].

Stem cells can be isolated from various tissues (e.g. embryonic, fetal, adult) that have different regenerative potential, a property that decreases with age [3]. Embryonic stem cells (ESCs), isolated from the inner cell mass of blastocysts, have been considered the gold standard for stem cell therapy because of their stronger plasticity and pluripotency; however, the clinical use of ESCs raises a number of issues [4]: 1) ESCs are not readily available and their collection requires the sacrifice of an embryo, a circumstance that gives rise to ethical concern and restriction policies; 2) after transplantation, ESCs usually form tumors or teratomas, and; 3) ESCs may not overcome the immunological incompatibility between the host and the donor. For these reasons, research has been focused on identifying new sources of stem cells that are abundantly available, easy to collect, safe for the host, and above any ethical concern. In 2006, the first preparation of induced pluripotent stem cells (iPSCs) was reported, where genetic reprogramming to an embryonic stem cell-like state was achieved by ectopic expression of four pluripotency genes [5]. These iPSCs did not overcome most of the disadvantages of ESCs, especially the low rate of reprogramming and the uncontrolled tumor formation and their immune tolerance is still debatable [6]. In contrast, amniotic-derived stem cells isolated from placenta respond favorably to all of these requirements. The placenta, normally discarded after delivery, is an extraordinary source of stem cells. In particular, the amniotic layer directly derived from the epiblast retains some of the characteristics of ESCs. The amniotic epithelial cells (AECs) express nuclear markers of pluripotency and surface markers of ESCs [7]. When grown in appropriate media, these cells can differentiate *in vitro* toward all the three germ layers [7] and they can rescue tissue and organ functions *in vivo* [8]. AECs are non-tumorigenic and do not have the ability to form teratomas when implanted in living animals [9]. Besides their regenerative functions, AECs combined a low immunogenicity with immunomodulatory and anti-inflammatory activities, thus allowing the transplantation under allo- and xenogenic settings [10]. In fact, AECs represent the first interface between the mother and the allogenic fetus, and play a crucial role in the feto-maternal immune tolerance [11].

As an organism ages, the individual cells in the body age as well [12]. This becomes even more evident *in vitro* when cultures of diploid human fibroblasts stop proliferating after a certain number of divisions as they reach the so-called “Hayflick limit” [13]. This process, called senescence, represents a permanent state of growth arrest, in which cells are still alive and metabolically active [14]. Many different mechanisms may account for the senescence phenotype, including telomere shortening, DNA damage, genome instability, mitochondrial dysfunction, and epigenetic modifications. It is widely accepted that senescence is a protective mechanism that cells mount to avoid malignant transformation, although it eventually ends up with an inflammatory phenotype that actually helps tumor progression [15]. It is unclear whether AECs provide protection against aging through the prevention of senescence-mediated inflammatory damage.

The present study was designed to investigate whether rat AECs retain multipotency, plasticity, and immune modulatory properties, and possess anti-proliferative activity against cancer cell lines as described with human [7, 16, 17], equine [18], and ovine [19, 20] AECs. We also investigated whether the conditioned medium (CM) of rat AECs contain soluble factors capable at improving markers of replicative senescence in human fibroblasts.

## RESULTS

### AECs retain stemness properties, low immunogenicity and show differentiation potential

**Figure 1:**
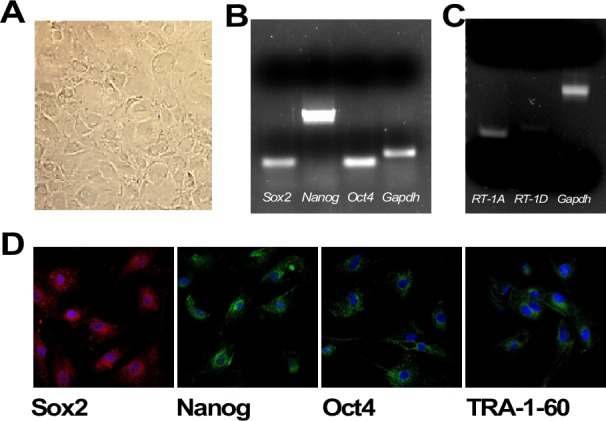
**A.** Plated rat amniotic epithelial cells (AECs) show the classical flat, epithelial phenotype (5x magnification). **B.** RT-PCR analysis of the pluripotent markers *Oct4*, *Nanog* and *Sox2*. **C.** RT-PCR of the MHC-I and MHC-II markers (*RT-1A* and *RT-1D*, respectively). **D.** Immunofluorescence of the pluripotency markers Sox2, Nanog, Oct4 and TRA-1-60. Nuclear counterstaining with DAPI is depicted in Blue (63x magnification).

AECs collected from rat amnion showed the classical flat, polygonal, and epithelial phenotype when maintained in tissue culture plates (Figure 1A). The markers of pluripotency Sox2 (SRY - Sex determining region Y- box 2), Nanog, and Oct4 (*Pou5f1*), were detected both at the mRNA and protein levels (Figure 1B and 1D). Sox2 was distributed both in the cytoplasm and nucleus, while Nanog and Oct4 were predominantly perinuclear. In contrast, the pluripotent stem cell marker TRA-1-60 (Tumor-related antigen-1-60) was expressed on the cell surface membrane (Figure 1D). The fact that rat AECs expressed low levels of *RT-1A* (homologous of MHC-I) and did not express *RT-1D* (homologous of MHC-II) (Figure 1C) indicate that these cells have retained low immunogenicity, as demonstrated in human AECs.

**Figure 2:**
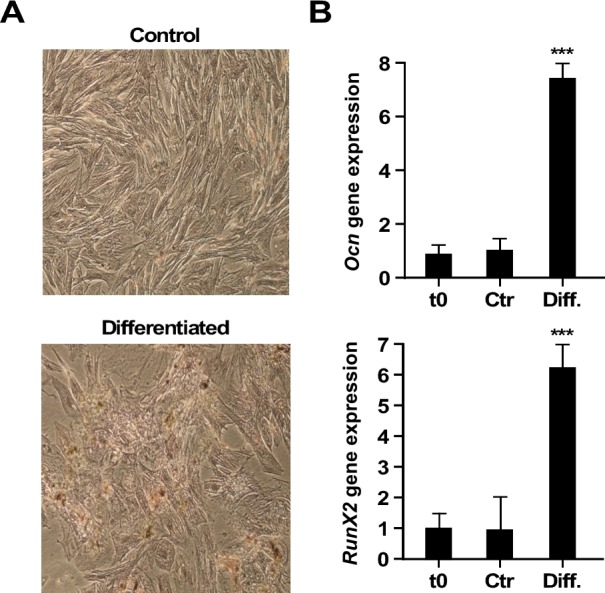
Osteogenic differentiation **A.** Alizarin Red Staining (10x). Upper row: control AECs; lower row: differentiated cells. **B.** Real-Time PCR of gene expression levels of osteogenic markers, *Ocn* and *Runx2*. t0, undifferentiated cells; Ctr, differentiation in control medium; Diff., Osteogenic differentiation medium. (***=*p* < 0.001). Shown is one representative of three independent experiments, each with triplicate samples.

Under osteo-inductive conditions, AECs changed morphology and accumulated calcium deposit (Figure 2A). A conversion toward osteoblast-related cell lines was observed, as evidenced by the up-regulation of *Ocn* (Osteocalcin) and *Runx2* (Runt related transcription factor 2) mRNAs (*p* < 0.001) (Figure 2B). The ability to differentiate rat AECs toward the osteogenic lineage confirms their plasticity.

### AECs modulate *Tnfa* mRNA production in activated macrophages

**Figure 3:**
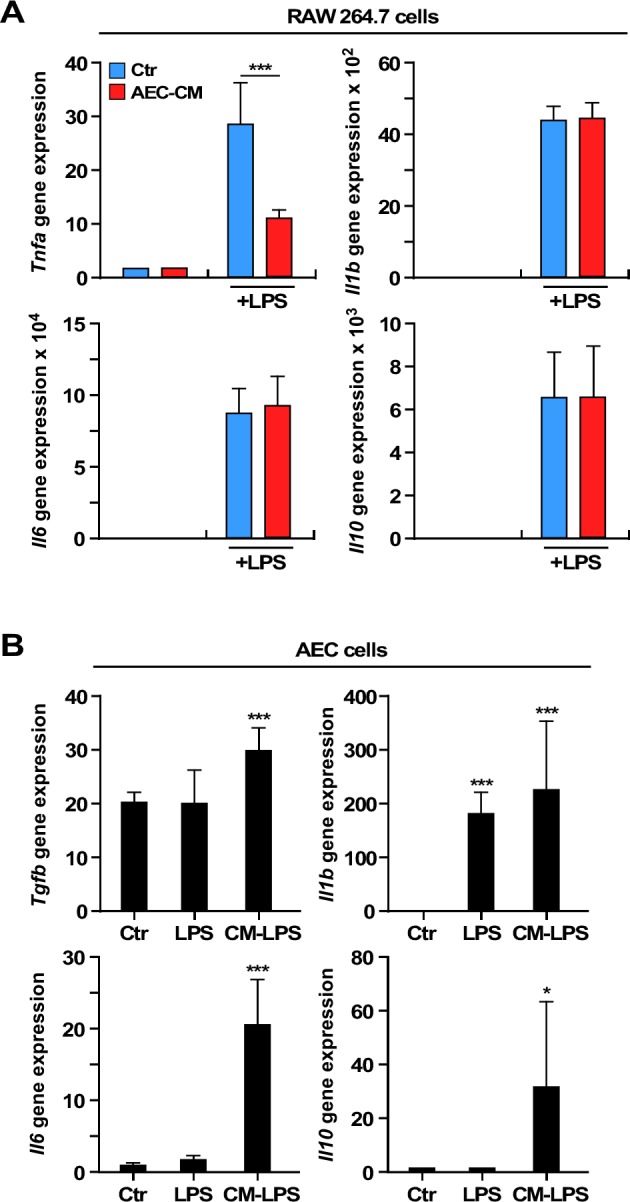
Expression of interleukins and cytokines mRNAs in RAW 264.7 and AEC cells **A.** *Tnfa* expression decreases in LPS-activated RAW 264.7 cells incubated with AEC-CM compared to Ctr medium. ***=*p* < 0.001. **B.** Expression of *Il10*, *Tgfb, ll6* and *Il1b* mRNAs increases in AECs incubated with the conditioned media of LPS-activated RAW 264.7 cells compared to cells in Ctr medium. *Il1b* is also induced by LPS alone. *=*p* < 0.05, ***=*p* < 0.001. Shown is mean and SD of three independent experiments, each with triplicate samples. Ctr= control medium, AEC-CM= AECs conditioned medium, CM-LPS=LPS-activated RAW 264.7 conditioned medium.

To investigate the immune modulatory properties of rat AECs, the behavior of AECs and RAW 264.7 (murine macrophages) was first studied by quantifying the mRNA expression levels of a panel of inflammatory cytokine genes. The levels of interleukin (*Il*)*10*, tumor necrosis factor (Tnfa), Il1b, and *Il6* mRNAs were very low when RAW 264.7 cells were exposed to 25 % conditioned media from AECs (AEC-CM) and control growth medium (Ctr) (Figure 3A). Next, the effect of AEC-CM on lipopolysaccharide (LPS)-activated RAW 264.7 cells was determined. LPS stimulation dramatically increased the expression of all four cytokines, but *Tnfa* mRNA levels were significantly lower in the presence of AEC-CM *vs*. Ctr medium (*p* < 0.001) (Figure 3A).

The incubation of AECs with LPS did not alter the expression of *Tgfb*, *Il6*, and *Il10* mRNAs, although the *Il1b* mRNA levels were induced in LPS-treated AECs (*p* < 0.001) (Figure 3B). However, treatment of AECs with the conditioned media of LPS-activated RAW 264.7 cells resulted in further increase in *Tgfb* (*p* < 0.001) and marked accumulation in *Il6* (*p* < 0.001) and *Il10* (*p* < 0.05) mRNAs (Figure 3B).

### AEC-CM inhibits tumor cell growth *in vitro*

The impact of AEC-CM on tumor cell growth was assessed in four different cancer cell lines using a colorimetric assay. HepG2 (human hepatocarcinoma cells), B16F10 (mouse skin melanoma), PANC-1 (human pancreatic carcinoma), and C6 (rat glioma) cells represent a good tumor sampling across tissues and species. The non-tumorigenic mouse embryonic fibroblasts (MEFs) were used as control for proliferation while the potency of AEC-CM was compared with MEF-CM.

**Figure 4:**
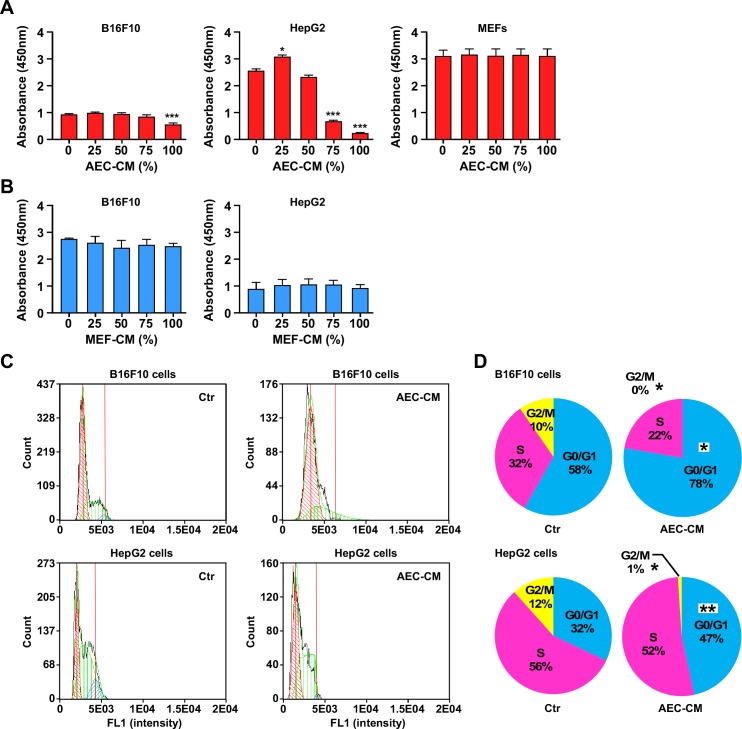
AEC-CM modulates tumor cell proliferation in a dose-response fashion **A.** B16F10, HepG2 and MEF cells were incubated in the presence of increasing amount of AEC-CM for 48 h following by the determination of cell proliferation using colorimetric assay. **B.** Proliferation of B16F10 and HepG2 cells is not affected using MEF conditioned medium (MEF-CM). ***=*p* < 0.001. Shown is mean and SD of three independent experiments, each with multiple replicate samples. **C.** Cell cycle analysis of B16F10 and HepG2 tumor cell lines after a 48h incubation with either Ctr medium or 100% AEC-CM. **D.** Quantitative assessment of cell distribution in each phase of the cell cycle. (*=*p* < 0.05, **=*p* < 0.01). Shown is one representative of two independent experiments, each with triplicate samples.

HepG2 cells were the most responsive to the anti-proliferative effects of AEC-CM, which occurred in a dose-dependent fashion (*p* < 0.001, both at 75% and 100% AEC-CM), whereas B16F10 cells were less responsive although still displaying a significant decrease in proliferation with 100% AEC-CM (*p* < 0.001) (Figure 4A). In contrast, MEFs proliferation was refractory to the addition of AEC-CM (Figure 4A) while PANC-1 and C6 cells followed the same pattern as HepG2 cells (Supplementary Figure 1A). None of the cell lines responded to MEF-CM treatment (Figure 4B and Supplementary Figure 1B).

To better understand the mechanisms involved in the anti-proliferative activity of AEC-CM, we performed apoptosis and cell cycle analyses. The rate of apoptosis was not significantly impacted in tumor cells and MEFs in response to AEC-CM treatment (data not shown); however, clear differences in cell cycle were observed where most of the AEC-CM-treated tumor cells (HepG2, PANC-1, B16F10) showed a significantly higher percentage stopped in G0/G1 checkpoint (*p* < 0.05) compared to cells incubated with Ctr medium (Figure 4C and 4D and Supplementary Figure 1C and 1D). MEFs were largely unresponsive to AEC-CM, in agreement with the proliferation assay.

### AECs inhibit tumor cell growth *in vivo*

**Figure 5:**
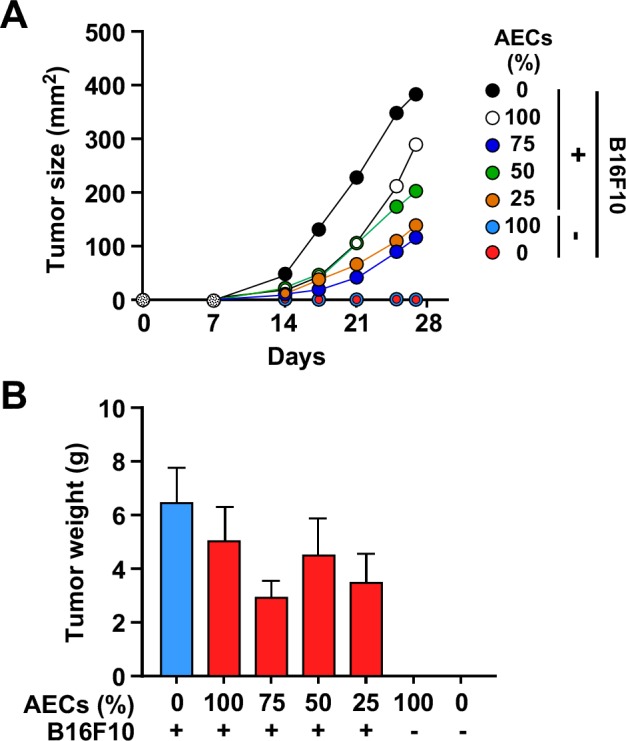
AECs slow tumor cell growth *in vivo* **A.** Mice were co-injected with 1×10^6^ B16F10 cells and increasing amounts (0.25 to 1×10^6^) of AECs, and tumor size was assessed for 25 days. (*=*p* < 0.05, **=*p* < 0.01, ***=*p* < 0.001). **B.** Weight of the explanted tumors between the experimental groups after 25 days (*n* = 6 animals per group). Although generally smaller, the tumors from AEC-injected mice did not show significant differences when compared to controls.

To confirm the results obtained in *vitro*, AECs were also tested *in vivo* by co-injecting them with B16F10 cells in C57BL/6J mice. A delay in tumor growth was apparent as evidenced by the significant reduction in tumor size using a caliper (*p* < 0.05) (Figure 5A). However, no statistically significant differences in tumor weight were observed at 25-day post injection, due to great variability among animals (Figure 5B and Supplementary Figure 2). Furthermore, there was no evidence of grafted AECs in the excised tumors (data not shown).

### AEC-CM does not affect cell proliferation, but delays senescence in human fibroblasts *in vitro*

**Figure 6:**
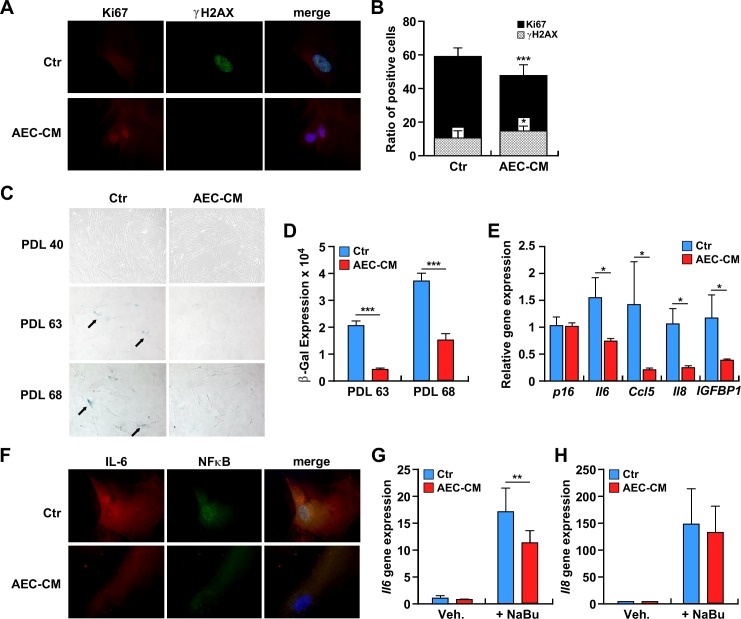
**A.** IMR-90 cells were incubated with Ctr medium or AEC-CM for ~100 days and levels of the proliferation marker Ki67 (red) and DNA damage marker γH2AX (green) were assessed (63x). **B.** Quantification of positive cells. (*=*p* < 0.05, ***=*p* < 0.001). Shown is one representative of two independent experiments, each with triplicate samples. **C.** IMR-90 cells at PDL 40, PDL 63, and PDL 68 were stained for SA β-gal. Note the higher SA β-gal staining when cells (at the same passage) were incubated with Ctr medium vs. AEC-CM. **D.** Quantification of SA β-gal staining. ***=*p* < 0.001. Shown is one representative of two independent experiments, each with triplicate samples. **E.** mRNA levels of *P16^ink4a^* and SASP cytokines in senescent IMR-90 cells incubated in Ctr and 20% AEC-CM for about 100 days. *IL6, CCL5, IL8 and IGFBP1* gene expression was significantly lower in cells treated with AEC-CM. *=*p* < 0.05. Shown is mean and SD of two independent experiments, each with triplicate samples. **F.** Expression and localization of IL6 and NF-κB in IMR-90 cells incubated with Ctr medium and AEC-CM for ~100 days. Nuclear counterstaining with DAPI is depicted in Blue. Shown is one representative of two independent experiments, each with triplicate samples. G and H. IMR-90 cells were incubated without or with 2 mM sodium butyrate (NaBu) in the presence of AEC-CM or Ctr medium to acutely induce senescence: **G.** *IL6* mRNA levels; **H.** *IL8* mRNA levels. **=*p* < 0.01. Shown is one representative of three independent experiments, each with triplicate samples.

The ability of AEC-CM to prevent the senescence of diploid human fibroblasts was tested in IMR-90 cells. These cells required approximately 70 population doubling levels (PDL), or around 120 days, to reach complete senescence. While the maintenance of IMR-90 cells in AEC-CM did not affect their proliferation rate, a trend toward longer survival was observed in IMR-90 cells supplemented with AEC-CM *vs*. Ctr medium (>70 *vs*. 68 PDL, *p* = 0.08) (Supplementary Figure 3). To better understand this phenomenon, late passage IMR-90 cells (PDL ~65) were analyzed for the proliferation marker Ki-67 and the DNA damage marker γH2AX. The results showed a higher percentage of Ki-67 positive cells and significantly lower γH2AX signal in IMR-90 cells maintained in AEC-CM *vs*. Ctr medium (*p* < 0.05 and *p* < 0.001, respectively) (Figure 6A and 6B).

### AEC-CM decreases markers of senescence

The senescence-associated β galactosidase (SA β-gal) staining was carried out to assess the extent by which IMR-90 cells were undergoing senescence. A low number of SA β-gal-positive cells was present during early passages, but at around PDL 63 the number of positive cells jumped significantly when IMR-90 cells were cultured in Ctr medium (*p* < 0.001) (Figure 6C and 6D). The SA β-gal staining was lower in response to AEC-CM supplementation and persisted with higher cell passages.

The senescence-associated cell cycle arrest is regulated mainly by the p16^ink4a^ pathway, whose induction leads to the activation of cyclin-dependent kinases. The gene expression of p16^ink4a^ (*CDKN2A*) was measured; however, no significant difference between the two experimental groups was observed (Figure 6E).

### AEC-CM decreases inflammation in senescent cells

Senescent cells increase endogenous production of a panel of inflammatory cytokines, termed SASP (Senescence-Associated Secretory Phenotype), among which IL6 is the most represented, along with chemokines and other soluble factors that can affect surrounding cells [21]. qPCR analysis showed a higher amount of *IL6, CCL5, IL8* and *IGFBP1* mRNA in cells maintained in Ctr medium *vs*. AEC-CM (*p* < 0.05) (Figure 6E). Furthermore, immunocytochemical analyses indicated the presence of a large pool of IL6 and NF-κB in the nuclear compartment of IMR-90 cells maintained in Ctr media, which was indicative of their activation state (Figure 6F). In contrast, cell treatment with AEC-CM reduced the amounts of IL6 and NF-κB, which were predominantly located in the cytoplasm (Figure 6F). To confirm the replicative senescence data, a new set of experiments was carried out where IMR-90 cells were treated with 2 mM sodium butyrate to induce acute senescence (data not shown). Sodium butyrate is a known histone deacetylase inhibitor capable of promoting cell cycle arrest [22]. Consistent with the reduced expression of SA β-gal staining in AEC-CM-treated IMR-90 cells (Figure 6C and 6D), the level of SASP proteins, especially *IL6*, was at least 1.5-fold lower than in IMR-90 cells incubated in Ctr medium (*p* < 0.01) (Figure 6G). *IL8* expression showed a similar trend, but the differences were not statistically significant (*p* = 0.68) (Figure 6H).

### Conditioned medium of AEC-CM treated IMR-90 cells slows the proliferation of tumor cells

**Figure 7:**
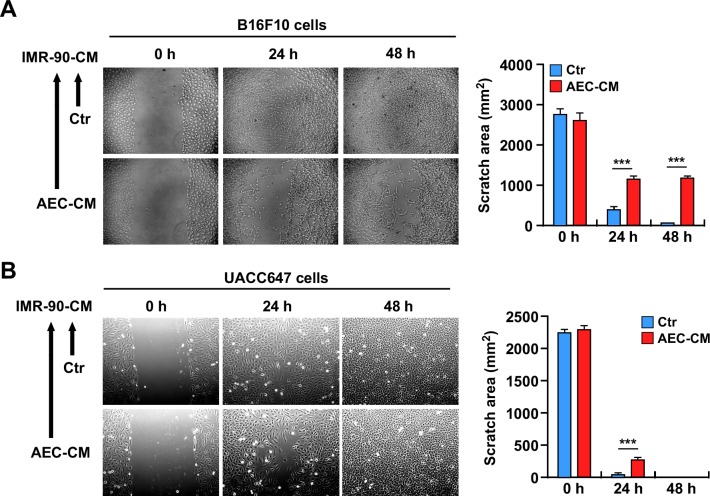
**A.** and **B.** Scratch test on B16F10 and UACC647 cells treated with conditioned medium from senescent IMR-90 cells incubated with either Ctr medium or AEC-CM during their lifespan. IMR-90 CM derived from AEC-CM-treated cells slow migration of B16F10 cells up to 48h, while the effect on UACC647 cells is most prominent in the first 24h. Shown is one representative of three independent experiments, each with triplicate samples. ***=*p* < 0.001.

Despite some controversies [23], few studies have indicated that senescent cells have the ability to increase tumor cell proliferation through their inflammatory secretome [15]. Therefore, the hypothesis that the secretome of AEC-CM-treated senescent IMR-90 cells is less prone to induce tumor progression was tested by performing *in vitro* scratch assays in murine B16F10 and human UACC647 melanoma cell lines supplemented with conditioned medium from senescent IMR-90 cells (Figure 7A and 7B). The results indicated a significant reduction in tumor cell migration when melanoma cells were incubated with conditioned media from IMR-90 cells pretreated with AEC-CM but not Ctr medium (*p* < 0.001) (Bar graphs depicted in Figure 7A and 7B).

## DISCUSSION

In this study, it was demonstrated that AECs isolated from rat term placenta retain some stemness properties and display *in vitro* differentiation potential and an immunoprivileged phenotype consistent with their physiological function during pregnancy. The conditioned medium from rat AECs contains soluble factors that confer immunomodulatory activity, are anti-proliferative in cancer cells, and delay the onset of senescence in a fibroblast cell line.

The placenta is a temporary organ that guarantees protection and nutrition to the developing fetus. The amnion represents the innermost layer of the placenta that encloses the fetus and protects it by regulating the composition and volume of the amniotic fluid [16]. Embryonic development differs between human and rodent. Amniogenesis is an early process in human: it is complete at day 10 when amnion becomes evident as a cavity into the epiblast cells [24]. The amnion in most mammals, including rodents, forms later during gastrulation. It appears as a simple bilayered membrane that remains intact during the whole gestation period surrounded by the visceral yolk sac, which probably fulfills the role of mechanical protection. In addition, rodent amnion, unlike that of human, does not fuse with chorion and it is not incorporated into the placenta [25]. For these reasons, the isolation procedure used here was slightly different from the one used for human protocol and was rather similar to that adopted for other animal models [26], whereby the yolk sac membrane was cut out and the inner white avascular membrane (amniotic membrane) collected. This method also differed from that adopted by Marcus et al. [27, 28] aimed at isolating mesenchymal cells (vimentin positive) from rat amniotic membrane.

Typically, stem cells express markers of self-renewal and pluripotency, especially Sox2, Nanog and Oct4. Oct4/Sox2 bind to the proximal region of the Nanog promoter and support its expression. The expression of Nanog is restricted to pluripotent cells, but it is down-regulated upon differentiation in a pattern that is well-conserved in mouse, rat, human [29] and sheep [19]. Here, gene and protein expression of the pluripotency markers confirmed stemness potential of rat AECs. The differentiation of stem cells toward the osteogenic lineage *in vitro* is the most common protocol used to demonstrate their plasticity [7, 18, 30, 31]. Following a 3-week differentiation protocol, rat AECs showed increased expression of osteoblast-related genes and were positive for calcium deposition. However, despite the similarities with ESCs, AECs cannot replace ESC or iPSC in stem cell biology, as their stemness properties are different.

Rat AECs conserved an immune-privileged phenotype and were able to modulate inflammatory cells that were activated *in vitro*. These innate immunological properties are in agreement with the physiological role of the amnion. The embryo represents a semi-allogenic graft for the mother because of the expression of paternal antigens, so natural mechanisms for avoiding rejection [11] entail an inflammatory response to implantation, followed by immunosuppression throughout the pregnancy [32]. The maternal immune system has developed systemic and local tolerance mechanisms: in the human placenta, expression of MHC class I is low and that of MHC class II is absent, but there is increased expression of the non-classical HLA-G that works as immunosuppressant during pregnancy [11, 33]. The rat *RT1-A* is a molecule with a highly polymorphic region harboring a MHC class Ia-like function that is expressed nearly ubiquitously and has high cell surface density. Moreover, the rat *RT1-D* gene is homologous to the MHC class II *HLA-DR* in humans [34, 35]. Because rat AECs were found to express low levels of *RT1-A* and did not express *RT1-D*, these cells may be useful in allo- and xenotransplantation, as the risk of rejection should be very low.

Human AECs exert immunomodulatory effects on lymphocytes by fostering the Th2 anti-inflammatory phenotype over Th1, both by acting directing with lymphocytes and through the conditioned medium, which contains secreted factors [36]. Amniotic cells dose-dependently inhibit the proliferation of activated peripheral blood mononuclear cells (PBMCs) [10, 19], but only few studies have focused on the effects of AECs on macrophages [37, 38, 39]. Like CD4^+^ lymphocytes, the macrophages polarize also toward different phenotypes in response to stimuli. Classically activated or M1 macrophages exhibit a Th1-like phenotype characterized by heightened inflammation, degradation of the extracellular matrix, and apoptosis. In contrast, alternatively activated or M2 macrophages display a Th2-like phenotype typified by enhanced extracellular matrix deposition, angiogenesis, and pro-reparative properties [40]. In the study herein, treatment of LPS-activated RAW 264.7 macrophages with AEC-CM led to a significant reduction in the release of *Tnfa*, but not *Il6* or *Il1b*. However, incubation of AECs with the conditioned media from LPS-activated RAW 264.7 cells triggered the production in *Il10*, *Tgfb* and *Il6* in AECs. These molecules are known M2 activators that signal through different pathways involving specific receptors [41]. Usually working as an inflammatory cytokine, IL6 can also have anti-inflammatory and pro-regenerative properties when it activates the classical signaling pathway (through both gp130 and IL6 receptor), compared to the trans-signaling mediated by the soluble form of IL6 receptor [42]. AECs express different Toll-like receptors (TRLs) on their surface, including TLR-4 [43], which could explain the inducible increase in *Il1b* mRNA levels in response to LPS activation. These data confirm that rat AECs possess immunomodulatory ability *in vitro* and shed further light on the mechanisms involved.

The conditioned medium from rat AECs contains soluble factors capable at inhibiting the proliferation rate of tumor cell lines from four different origins, in agreement with earlier reports using human AECs [44, 45]. Rat AEC-CM was very effective at reducing HepG2 cell proliferation while exhibiting lower potency, but still significant, against PANC-1, C6, and B16F10 tumor cells. Some cancer cell lines are also more sensitive than others to the inhibitory effects of human AECs [46]. Here, further investigation into the mechanisms implicated in the antiproliferative effects of AEC-CM revealed the presence of soluble factors that interfere with cell cycle controls in tumor cells [47]. Indeed, an increase in the number of cells blocked in the G0/G1 phase of the cell cycle was observed with most of the cell lines tested (HepG2, B16F10 and PANC-1), in agreement with a previous study [44], while C6 cells were mostly accumulating in the S phase in response to AEC-CM treatment.

AECs were also able to delay the growth of B16F10 tumors *in vivo*, although the mice eventually developed tumors with similar final tumor weights as mice injected with B16F10 cells alone. Even though no grafted AECs —pre-loaded with Qtracker fluorescent particles— were found in the excised tumors, the fact that co-injection of AECs with malignant B16F10 cells caused a lag in the exponential tumor growth is encouraging and suggests a significant inhibition in proliferative cell population during early stages of tumor development.

Although the precise factors and mechanisms responsible for the immunoregulatory roles of AECs remain unknown, previous studies have reported that IL6, IL10, IL1β and TGFβ, along with prostaglandin E2 (PGE2), indoleamine 2,3-dioxygenase (IDO), and HLA-G are constitutively secreted mediators that confer the anti-inflammatory properties of AECs [48]. Our experiments, however, cannot exclude that some of the effects are due to exosomes rather than soluble factors. In fact, exosomes and microvescicles (MV) can be released by AECs and be involved in anti-inflammatory and anti-proliferative responses. A recent study from Lange Consiglio [49] suggested that AECs secrete MVs, but these were only responsible for the down-regulation of some metalloproteinases and *TNFα* expression in primary LPS activated-tenocytes, but not for inhibition of PBMC proliferation *in vitro*, contrary to the whole conditioned medium collected from the same cells. Further studies are required to better assess this point.

The ability of rat AECs to delay cellular senescence *in vitro* is of significance. Cell senescence is one of the mechanisms implicated in cellular and organismal aging. The targeting of senescent cells contributes to increased lifespan and healthspan partly by preventing the chronic inflammatory response that triggers tumor growth [12,50]. Activation of the DNA damage response pathway is accompanied by cellular senescence that is characterized by increased expression of the tumor suppressors p53-p21 and/or activation of p16^ink4a^/RB and subsequent inhibition of cyclins and cyclin-dependent kinases (CDKs), leading to cell cycle arrest [12]. A second mechanism involved in the establishment of cellular senescence is the formation of SAHF. These regions of highly condensed chromatin sequester genes implicated in cell cycle regulation and senescence-associated DNA-damage foci (SDFs), which harbor histone modifiers such as γH2AX phosphorylation [51]. In this study, the long-term influence of AEC-CM on the inducible senescence of IMR-90 cells was assessed. The proliferation rate of IMR-90 cells was refractory to AEC-CM treatment and these cells ultimately proceeded toward a permanent state of cell cycle arrest [a.k.a. senescence] at the same rate as IMR-90 cells maintained in Ctr medium. Furthermore, expression of *p16^ink4a^* mRNA was unchanged upon AEC-CM treatment, indicating stabilization of this cell cycle inhibitor protein [52]. Compared to IMR-90 cells in Ctr medium, cells incubated with AEC-CM exhibited lower SA β-gal staining and SAHF formation, and the shape of the nucleus appeared regular. Persistent DNA damage signaling triggers the secretome of senescent cells [53], which consists of inflammatory factors collectively known as SASP, a process that is independent of p16^ink4a^ activation [54]. Here, AEC-CM treatment of IMR-90 cells significantly reduced expression of several markers of senescence, including *IL6, CCL5, IL8* and *IGFBP1* [21], whose regulation is essentially at the mRNA level [55]. NF-κB is a master regulator of inflammatory response and SASP [56] by acting through transcription of a plethora of inflammatory cytokines, including IL6. The nuclear localization of NF-κB in IMR-90 cells incubated with Ctr medium was consistent with a senescence-inducible pro-inflammatory state, a circumstance not found in AEC-CM-treated cells. Furthermore, the fact that IMR-90 cells incubated with AEC-CM slowed the proliferation and migration of tumor cells further confirms that AEC-CM diminished the secretion of inflammatory mediators by senescent cells when compared to Ctr medium.

These properties justify the increasing attention that AECs have received recently. Even if their therapeutical properties were already known since the last century, the mechanisms related to their regenerative role have only been studied in the last decades. The first attempts of using placenta for regenerative purposes were aimed especially at skin reconstruction. Its clinical success was ascribed not only to the enhanced wound-healing properties, but also to the lower inflammatory response that cells or amniotic membranes offered when compared to other available sources. As the signaling mechanisms involved in the innate immunomodulatory activity and anti-inflammatory properties of AECs have been largely elucidated, there is a momentum toward the use of AECs in the resolution of inflammatory diseases [57, 58, 59, 60].

The anti-senescence effects of AECs combined with other recent evidences on the utilization of AEC transplantation to extend lifespan in animal models [61, 62] open new frontiers in the use of fetal stem cells in the aging field. AEC-CM delays the onset of senescence without extending the lifespan of cells, possibly because the AEC secretome impedes the formation of inflammation-associated processes that induce senescence. Earlier reports have indicated delayed senescence in normal fibroblasts using serum from calorie restricted animals [63], rapamycin [64], and resveratrol [65] treatment. These findings open new perspectives for the use of AECs in regenerative medicine.

Although the pre-clinical use of human amniotic stem cells is routinely performed in animal models [66], rat AECs may provide a new valuable tool in autologous and allogeneic transplantation settings in rodent models, as both approaches are required to overcome interspecies rejection, which happens occasionally when human cells are implanted in animals. Moreover, the use of rat AECs may allow the study of cell-induced regenerative mechanisms into an immunocompetent animal with a spontaneous/inducible disease or in which a tissue-specific defect has been experimentally produced.

In conclusion, rat AECs retain the characteristics of multipotency and differentiation potential. They have immunological, anti-inflammatory and anticancer properties, as already described in human and large mammals. Because of these properties, rat AECs may have a protective role in conditions involving inflammatory processes, such as in aging and reparative phases and, thus, represent a promising tool for regenerative medicine.

## MATERIALS AND METHODS

### Isolation of AECs and production of conditioned medium (AEC-CM)

Twenty uteri from 18-day pregnant rats were kindly provided by Dr. Mark Mattson (NIA). Uteri were dissected under a stereomicroscope and the white, avascular amniotic layer was collected and extensively washed in PBS. Next, the membranes were treated with 0.25% trypsin-EDTA (Sigma-Aldrich) for 30 min in a humidified incubator with 5% CO_2_ at 37°C. Tissues were then filtered through a 70μm cell strainer to collect single cell suspension and centrifuged at 1500 rpm for 5 min. The cell pellet was resuspended in complete growth medium [DMEM, 10% FBS and 1% PenStrep (all from Gibco), also termed ‘control medium’], and the number of cells counted before being immediately frozen. Each utero averaged 10 to 12 amnia that allowed the collection of about 6×10^6^ cells/animal.

The preparation of AEC-CM was carried out as followed: 0.5×10^6^ AECs were seeded in a 175cm^2^ flask and maintained in control (Ctr) medium. Medium was changed once after complete cell adhesion and AEC-CM was collected 48 h later while cells were growing exponentially. AEC-CM was centrifuged at 5000 x g at room temperature for 10 min, filtered through a 0.2 μm membrane, aliquoted, and then frozen at −80°C. Unless indicated otherwise, 20% AEC-CM was used for chronic treatments (senescence) while acute treatments required 25% AEC-CM.

### RNA extraction and gene expression analysis

Total RNA was extracted from AECs using RNeasy mini kit (Qiagen) according to the manufacturer's specifications. RNA concentration and purity were measured uisng Nanodrop Spectrophotometer (Nanodrop^®^ ND1000, Thermo Scientific). One μg of RNA was reverse transcribed into cDNA using iScript Advanced cDNA Synthesis Kit for RT-qPCR (BioRad) and incubated for 30 min at 42°C, followed by 5 min at 85°C. To analyze markers of stemness and MHC, End-Point PCR was performed with iProof DNA High fidelity DNA polymerase Kit (BioRad) according to the manufacturer's protocol under the following conditions: Initial denaturation at 98°C for 30 s, 35 cycles at 98°C for 10 s (denaturation), 60°C for 30 s (annealing), 72°C for 30 s (elongation), and a final elongation at 72°C for 10 min. Ten μl of PCR products were run on a 2% agarose gel and the amplifications bands were visualized with a GelDoc Imager (Biorad). Real-time RT-PCR was performed using iTaq universal SYBR^®^ Green Supermix (BioRad) under the following conditions: 95°C for 30 s, 40 cycles at 95°C for 3 s, and 60°C for 30 s using a StepOne Plus Real Time PCR system (Applied Biosystems). Primers used are listed in Supplementary Table 1. Fold changes in gene expression were measured using the 2^−ΔΔCT^ method.

### Immunocytochemistry

Cells were seeded at 1×10^3^ cells/cm^2^ in 35-mm glass bottom culture dishes (MatTek Corp.) and were allowed to grow until reaching ~70% confluence. After a series of PBS washes, cells were fixed in 4% paraformaldehyde (PFA, Electron Microscopy Sciences) for 15 min prior to permeabilization with PBS containing 0.1% Triton X-100 (Sigma-Aldrich) for 10 min. Cells were blocked with 10% donkey serum for 1 h and then primary antibodies against various targets at specific dilutions (listed in Supplementary Table 2) were added for an overnight incubation at 4°C. Immediately after 3 washes in PBS supplemented with 0.05% Tween-20 and 1% donkey serum, cells were incubated with secondary antibodies for 1 h at room temperature. After 3 more washes, samples were treated with Vectashield Mounting medium (Vector laboratories) containing DAPI (4′,6-diamidino-2-phenylindole) to stain for nuclei. Images were acquired with a Zeiss LSM 710 confocal microscope.

### *In vitro* osteogenic differentiation

AECs were plated at 3×10^3^/cm^2^ in 35-mm culture dishes and were allowed to grow until reaching 80% confluency, at which point the growth medium was switched to the differentiation medium consisting of Ctr medium supplemented with 0.1 μM dexamethasone, 50 μM ascorbic acid, and 10 mM β-glycerophosphate (all from Sigma-Aldrich). Cells were grown for 3 weeks with medium change twice weekly. The degree of osteogenic differentiation was determined by gene expression analysis of *Ocn* and *Runx2*, and was further confirmed by Alizarin Red S staining as followed: After a series of PBS washes, cells were fixed (in 4% PFA for 15 min) prior to staining with 40 mM Alizarin Red (Santa Cruz Biotechnologies), pH 4.2, for 30 min under gentle agitation. Images were acquired with a Zeiss Axiovert 200 microscope.

### Immunomodulation assay

RAW 264.7 murine macrophages were seeded at 1×10^4^ cells/cm^2^ in a 12-well plate and were allowed to grow in DMEM complete medium (10% FBS and 1% PenStrep) for 24 h. Then, cells were treated without or with 1μg/ml LPS (E. Coli O111:b4, Calbiochem) in Ctr medium supplemented or not with 25% AEC-CM. After 6 h of incubation, cells were collected for the measure of *Tnfa*, *Il6, Il10*, and *Il1b* mRNA levels. In a second set of experiments, AECs were incubated with the conditioned medium of LPS-treated RAW 264.7 cells or LPS alone for 6 h. The AECs were collected for the measure of *Tgfb, Il6, Il10*, and *Il1b* mRNA levels.

### Tumor cell proliferation, apoptosis and cell cycle analysis

HepG2, PANC-1, B16F10 and C6 tumor cell lines (ATCC) were seeded in a 96-well plate at 5×10^3^ cells/well in DMEM complete medium supplemented with increasing amount (0, 25%, 50%, 75% or 100%) of either AEC-CM or MEF-CM, the latter conditioned medium derived from MEFs. Forty-eight hours later, 10 μl of CCK-8 solution (Dojindo Molecular Technologies, Inc.) was added to each well for 3 h before measuring the absorbance at 450 nm using a microplate spectrophotometer (Biorad).

For the apoptosis and cell cycle assays, tumor cells were seeded in 6-well tissue culture plates with Ctr medium and AEC-CM. After one day, apoptosis was induced with 100 nM etoposide (Sigma) for 24 h after which cells were detached with 0.05% trypsin, spun down for 5 min at 400 x g and resuspended in PBS. Aliquots of 1×10^5^ cells were collected from each sample and resuspended in 40 μl of Annexin V Binding Buffer, 5 μl Annexin V-FITC, and 5 μl propidium iodide (PI) solution (all from Nexcelom). After incubation for 15 min at RT, cells were washed in PBS, centrifuged at 400 x g for 5 min and resuspended in Annexin V Binding Buffer. For the cell cycle assay, cells were collected after 48h, fixed in 70% ice-cold ethanol in PBS, incubated on ice for 15 min, spun down at 300 x g for 8 min and resuspended in 150 μl of PI/RNase staining solution (Nexcelom). After 40 min incubation at 37°C, cells were washed in PBS. Both the Annexin V+PI and CBA-cell cycle PI660nm assays were run using a Cellometer Vision CBA (Nexcelom) [67] and the results were analyzed with FCS Express 4 software (De Novo Software).

### *In vivo* tumor growth inhibition

Forty-two male C57BL/6J mice were single-housed in duplex caging in a room maintained at a constant temperature (20-22°C) and humidity (30-70%) in a light:dark 12:12-h schedule, according to established animal protocols and NIH guidelines. AECs were freshly collected and expanded *in vitro* for 48 h, then were detached with 0.05% trypsin, washed with PBS and counted. AECs and B16F10 cells were pre-labeled with Qtracker^®^ Cell Labeling kit (Gibco, Life Technologies) by incubating cells with the labeling solution (655 for AECs and 525 for B16F10 cells) for 60 min at 37°C. After a series of washes, cells were ready for injection in mice.

Six mice per group were injected in the lower back with either PBS, 1×10^6^ B16F10 melanoma cells or 1×10^6^ AECs alone as controls, and 1×10^6^ B16F10 cells with increasing amounts of AECs (0.25×10^6^, 0.5×10^6^, 0.75×10^6^ and 1×10^6^) as treatment groups. Tumor growth was checked twice a week and tumor size was measured with a caliper. After 25 days, when the first tumors reached 20×20 mm^2^, animals were euthanized by cervical dislocation. Tumors were excised and size and weight were recorded. Tumors were then fixed in 10% neutral buffered formalin (Thermo Scientific) for 48 h, dehydrated in 30% sucrose (Sigma) for 48 - 72 h (depending on the tumor size), frozen and cut to 10 μm thickness. Specimen were imaged with a Zeiss Axiovert 200 microscope.

### Senescence studies

IMR-90 cells are normal human diploid fibroblasts (I90-79, Coriell Institute for Medical Research) that can undergo senescence both by replicative exhaustion and acute stress. For the replicative exhaustion study, 1×10^5^ IMR-90 cells were seeded in 60 mm plates and were allowed to grow in Minimum Essential Medium (MEM), 10% FBS and 1% PenStrep, supplemented with either 20% AEC-CM or Ctr medium and split when close to confluency. Cells were then detached with 0.05% trypsin-EDTA and counted using a cell counter (Cellometer Vision CBA, Nexcelom). The number of doublings for each passage was calculated by the formula: *N* = 3.32 [log (total viable cells at harvest / total viable cells at seeding)].

For the stress-induced senescence experiment, IMR-90 cells were seeded at 5×10^4^ cells/well in 12-well plates and treated with 2 mM sodium butyrate (Sigma-Aldrich) for 48 h in the presence of 20% AEC-CM or Ctr medium.

Senescence was measured by three different methods: β-gal staining, Ki67 and γH2AX levels and RT-qPCR analysis of genes associated with SASP (as specified in Supplementary Table 1). The senescence-associated β-gal staining was performed using a commercial kit (Cell Signaling). The number of SA β-gal positive cells was counted with a Zeiss Axiovert 200 microscope while the stain intensity was quantified using ImageJ software.

### Scratch test for tumor cell proliferation

Scratch test was performed following the protocol of Liang *et al.* [68]. Briefly, B16F10 and UACC647 cells were seeded in 6-well plates (Corning) and were allowed to grow in DMEM complete medium until reaching confluency. Then, a scratch was made in the plate using a p200 pipet tip, and each plate was marked to have a reference point. Pictures were taken in the same field every 24 h with a Zeiss Axiovert 200 microscope. The rate of wound closure was performed with ImageJ software (NIH).

### Statistical analysis

Statistical analysis was performed using Prism 5 (Graph Pad software). Multiple comparisons were analyzed by one-way ANOVA, followed by Tukey's post hoc test. Senescence curves, SASP gene expression and cell cycle were analyzed by *t*-test. Data are expressed as means ± standard deviation (SD), with *p* value of ≤ 0.05 considered statistically significant.

## SUPPLEMENTARY MATERIAL FIGURES AND TABLES


