# Supplementary material for: Conditioned medium derived from rat amniotic epithelial cells confers protection against inflammation, cancer, and senescence
Source: Oncotarget. 2016 May 29;7(26):39051–64. doi: 10.18632/oncotarget.9694 (PMC5129913; doi:10.18632/oncotarget.9694)
Supplement: Supplementary file 1 [file oncotarget-07-39051-s001.pdf]

Conditioned medium derived from rat amniotic epithelial cells confers protection against inflammation, cancer, and senescence

Supplementary Material

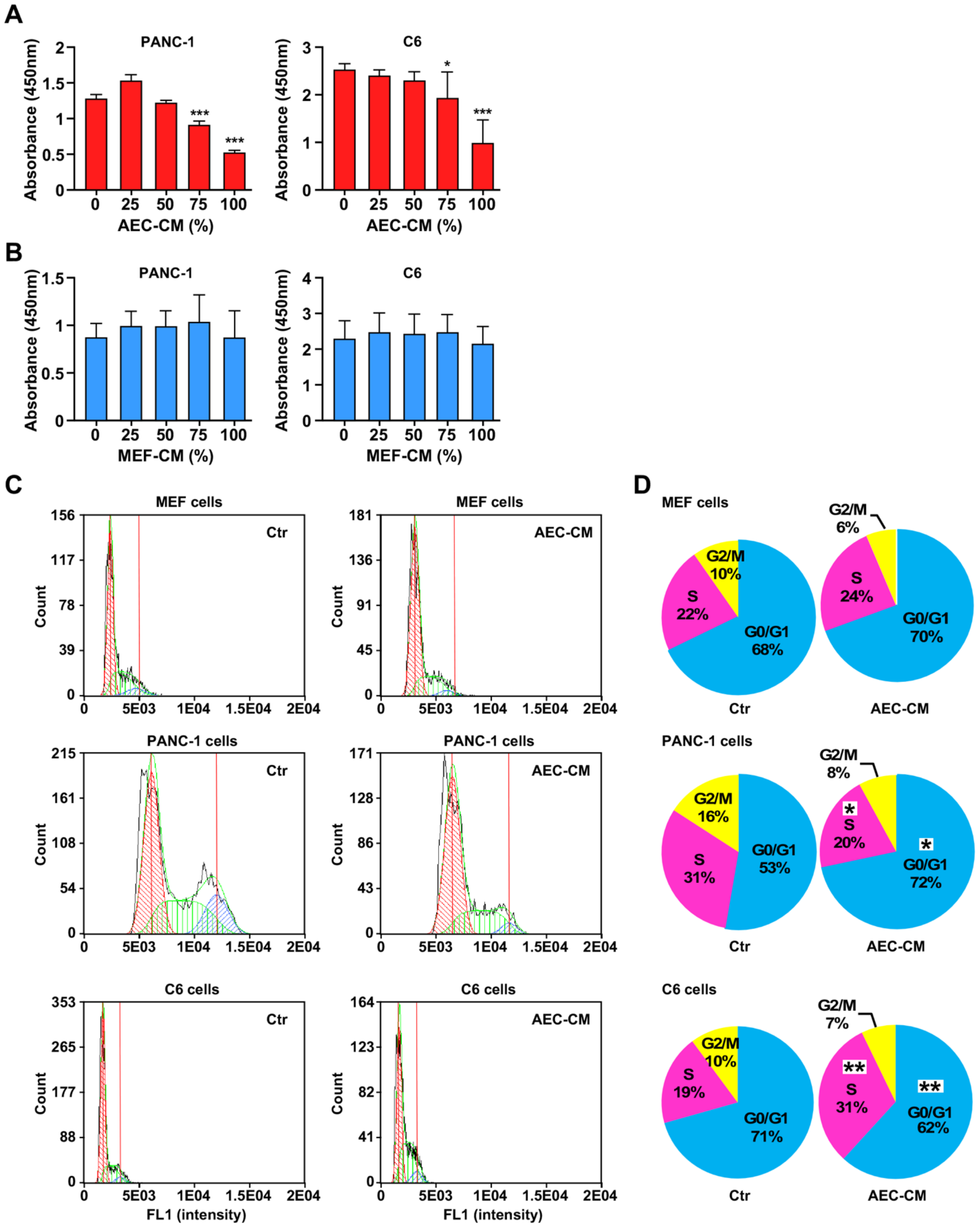

Supplementary Figure 1. AEC-CM modulates tumor cell proliferation. PANC-1 and C6 cells were incubated with increasing concentrations of AEC-CM (panel **A**) and MEF-CM (panel **B**)

for 48 h followed by the determination of cell proliferation using a colorimetric assay. **C.** Cell cycle analysis of MEFs, PANC-1 and C6 cell lines after supplementation with Ctr and 100% AEC-CM. Shown in representative of two independent experiments, each performed with triplicate samples. **D.** Quantitative analysis of the number of cells in each cell cycle checkpoint. *, $p<0.05$; **, $p<0.01$ *versus* Ctr.

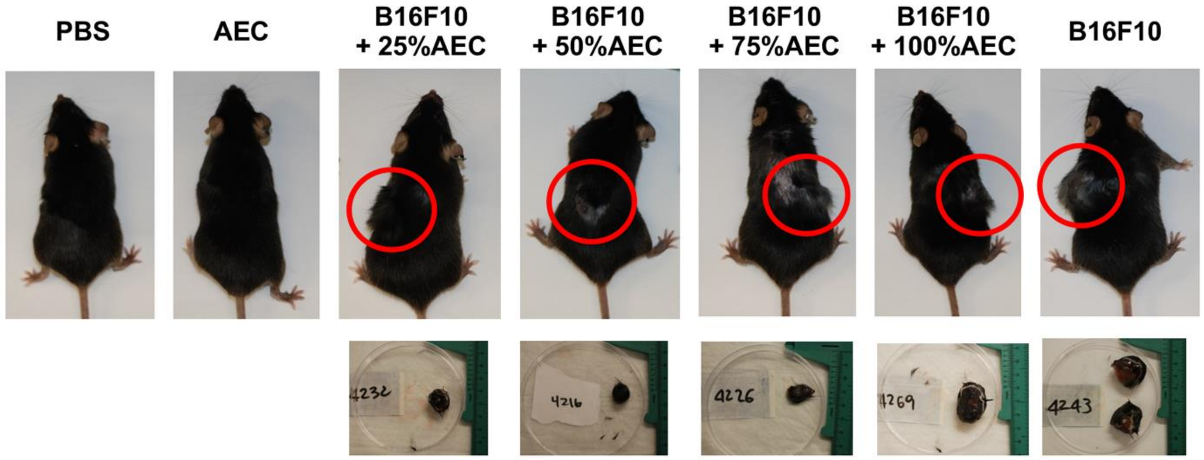

Supplementary Figure 2. Co-injection of AECs slows B16F10 tumor growth in C57BL/6J mice. When compared to mice injected with B16F10 alone, co-injection of varying amounts of AECs (100% = $1x10^6$ cells) with B16F10 cells reduces tumor size. N=6 per group.

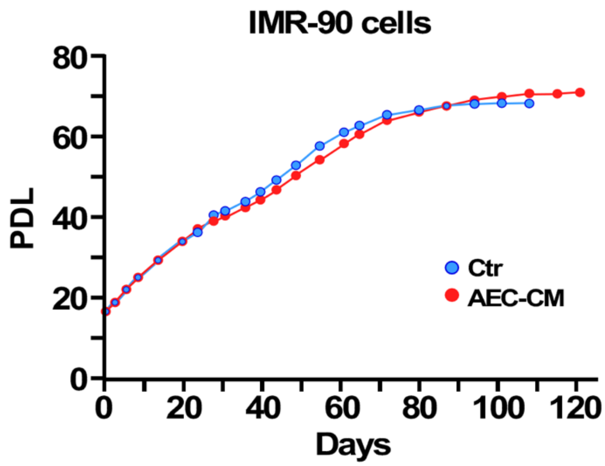

Supplementary Figure 3. AEC-CM treatment does not alter the proliferation rate of IMR-90 cells. Growth curves of human IMR-90 fibroblasts supplemented with Ctr and AEC-CM (20%). PDL, population doubling level.

Supplementary Table 1. List of primer sequences used for quantitative PCR.

r Oct4 Fw	CCCCATTTCACCACACTCTACTC
r Oct4 Rv	GTGACAGGAACAGAGGGAAAGG
r Nanog Fw	GCCCTGAGAAGAAAGAAGAG
r Nanog Rv	CTGACTGCCCCATACTGGAA
r Sox2 Fw	CTGTTTTTTCATCCCAATTGCA
r Sox2 Rv	CGGAGATCTGGCGGAGAATA
r GAPDH Fw	CAGGGCTGCCTTCTCTTGTG
r GAPDH Rv	CTTGCCGTGGGTAGAGTCAT
r RT1-A Fw	ATCACCCGGAACAAGTGGGA
r RT1-A Rv	ATTCAACTGCCAGGTCAGGG
r RT1-D Fw	AACGGACAACCTGTCACCAA
r RT1-D Rv	GATGAGCACAATCCCGACGA
r GAPDH Fw	ACCACAGTCCATGCCATCAC
r GAPDH Rv	TCCACCACCCTGTTGCTGTA
r Ocn Fw	TATGGCACCACCGTTTAGGG
r Ocn Rv	CTGTGCCGTCCATACTTTCG
r Runx2 Fw	CAACCACAGAACCACAAGTGC
r Runx2 Rv	CACTGACTCGGTTGGTCTCG
m TNFα Fw	CCCTCACACTCAGATCATCTTCT
m TNFα Rv	GCTACGACGTGGGCTACAG
m IL10 Fw	TAACTGCACCCACTTCCCAG
m IL10 Rv	AAGGCTTGGCAACCCAAGTA
m IL1β Fw	GCAACTGTTCCTGAACTCAACT
m IL1β Rv	ATCTTTTGGGGTCCTTCAACT
m IL6 Fw	GACAAAGCCAGAGTCCTTCAGA
m IL6 Rv	GTCTTGGTCCTTAGCCACTCC
m GAPDH Fw	GGGTTCCTATAAATACGGACTGC
m GAPDH Rv	TCTACGGGACGAGGCTGG
h CXCL8 Fw	GGTGCAGTTTTGCCAAGGAG
h CXCL8 Rv	TTCCTTGGGGTCCAGACAGA
h IL6 Fw	ACCCCCAATAAATATAGGACTGGA
h IL6 Rv	TTCTCTTTCGTTCCCGGTGG
h IGFBP1 Fw	TCACAGCAGACAGTGTGAGAC
h IGFBP1 Rv	CCATTCCAAGGGTAGACGCA
h Ccl5 Fw	CAGTCGTCCACAGGTCAAGG

h Ccl5 Rv	CTTGTTCAGCCGGGAGTCAT
h p16ink4 Fw	ACTTCAGGGGTGCCACATTC
h p16ink4 Rv	CGACCCTGTCCCTCAAATCC
h GAPDH Fw	CTCTGCTCCTCCTGTTCGAC
h GAPDH Rv	GCGCCCAATACGACCAAATC
r TGFb Fw	AGGGCTACCATGCCAACTTC
r TGFb Rv	CCACGTAGTAGACGATGGGC
r IL10 Fw	CCTCTGGATACAGCTGCGAC
r IL10 Rv	GTAGATGCCGGGTGGTTCAA
r IL6 Fw	AGCCAGAGTCATTCAGAGCAA
r IL6 Rv	ATTGGAAGTTGGGGTAGGAAG
r IL1β Fw	GACTTCACCATGGAACCCGT
r IL1β Rv	GGAGACTGCCCATTCTCGAC

Supplementary Table 2. List of commercial antibodies used for confocal microscopy.

SOX-2 (1:100) Santa Cruz Biotech. [sc-17320]	Anti-goat Alexa564 (1:500) Sigma
Nanog (1:50) Abcam [ab106465]	Anti-rabbit Alexa488 (1:500) Sigma
OCT-4 (1:100) Santa Cruz Biotech.[sc-5279]	Anti-mouse Alexa488 (1:500) Sigma
TRA-1-60 (1:100) Cell Signaling [#4746]	Anti-mouse Alexa488 (1:500) Sigma
NF-κB p65 (1:100) Abcam [ab32536]	Anti-rabbit Alexa488 (1:500) Sigma
IL-6 (1:50) R&D System [AF-206-NA]	Anti-goat Alexa564 (1:500) Sigma
Gamma H2AX(1:1000) Abcam [ab81299]	Anti-rabbit Alexa488 (1:500) Sigma
Ki-67 (1:100) Santa Cruz Biotech. [sc-23900]	Anti-mouse Alexa564 (1:500) Sigma
